# Multi-Sensor Consensus Estimation of State, Sensor Biases and Unknown Input

**DOI:** 10.3390/s16091407

**Published:** 2016-09-01

**Authors:** Jie Zhou, Yan Liang, Feng Yang, Linfeng Xu, Quan Pan

**Affiliations:** Key Laboratory of Information Fusion Technology, Ministry of Education, School of Automation, Northwestern Polytechnical University, Xi’an 710129, China; zhoujie@mail.nwpu.edu.cn (J.Z.); yangfeng@nwpu.edu.cn (F.Y.); xulinf@gmail.com (L.X.); quanpan@nwpu.edu.cn (Q.P.)

**Keywords:** bias estimation, state estimation, sensor registration, network consensus

## Abstract

This paper addresses the problem of the joint estimation of system state and generalized sensor bias (GSB) under a common unknown input (UI) in the case of bias evolution in a heterogeneous sensor network. First, the equivalent UI-free GSB dynamic model is derived and the local optimal estimates of system state and sensor bias are obtained in each sensor node; Second, based on the state and bias estimates obtained by each node from its neighbors, the UI is estimated via the least-squares method, and then the state estimates are fused via consensus processing; Finally, the multi-sensor bias estimates are further refined based on the consensus estimate of the UI. A numerical example of distributed multi-sensor target tracking is presented to illustrate the proposed filter.

## 1. Introduction

The well-known Kalman filter is optimal given a linear Gaussian model, but its performance will deteriorate in the presence of unknown biases, such as the unknown and time-varying delays in chemical processes [1,2,3,4], faults or failures in fault-tolerant diagnosis and control systems [5,6], registration errors in multi-sensor fusion [7,8,9,10], or inertial drift in navigation [11,12,13,14]. In general, such a bias is represented as an unknown input (UI) to the nominal model. To the best of our knowledge, approaches to UI modeling and the design of corresponding filters typically fall into one of the following categories.

The first type of UI is zero-mean random noise with unknown covariance. In the case of the stationary noise processes in linear dynamic systems, the covariance can be identified via Bayesian or maximum likelihood estimation. A corresponding filter has been applied for orbit determination for near-earth satellites [15,16]. Recently, this type of filter design has been extended to time-varying covariance [17] and jump Markov stochastic systems [18]. Furthermore, an M-robust estimator [19] has been derived for the simultaneous adaptive estimation of unknown states and observation noise statistics. An auto-covariance least-squares method [20] has been presented to achieve lower-variance covariance estimates along with the necessary and sufficient conditions for the uniqueness of the estimated covariances. By treating unknown covariances as missing data, the adaptive estimation problem has been transformed into a problem of the joint optimization of state estimation and parameter identification, allowing it to be solved in an expectation-maximization (EM) iterative processing framework [21].

The second type of UI is unknown but deterministic. Using least-squares estimation and moving-window hypothesis testing, the corresponding filters can cope with cases in which the UI is piecewise constant [22] or a sum of basis functions with piecewise-constant weights [23]. The problem of state estimation with UIs in both dynamic and measurement models has been addressed using a joint EM optimization scheme for state estimation, parameter identification and iteration termination decision-making [24], and this approach has been extended to a distributed EM algorithm for sensor networks [25], and further extended to an adaptive divided difference filter for nonlinear system with multiplicative parameters [26].

The third type of UI is completely arbitrary, without the availability of any prior knowledge about its evolution. An asymptotically stable and UI-decoupled observer has been derived [27] based on the condition that the rank of its distribution matrix must be less than that of the UI. For the case in which the UI appears only in the process equation, a combination of least-squares estimation and the Kalman filter has been proposed for joint state and UI estimation, and the necessary and sufficient condition for the existence of such a joint estimator has also been presented [28]. In addition, this result has been extended to the case in which the UI appears in both the process and measurement models [29].

The fourth type of UI is norm-bounded, and robust offline filters have been designed to minimize the gain of the transfer function from the UI to the estimation error [30]. As an alternative approach, a method of linear minimax regret estimation has been developed to minimize the worst-case regret over all bounded data [31].

The fifth type of UI is characterized by a randomly switching parameter obeying a known Markov chain. The corresponding solutions fall within the scope of multiple model estimators [32,33,34,35,36,37], such as the interacting multiple model (IMM), which is well known in maneuvering-target tracking. Motivated by the concept of establishing a general framework for joint state estimation and data association in clutters for the tracking of a maneuvering target, the linear minimum-mean-square-error (MMSE) estimator has been derived for discrete-time Markovian jump linear systems with stochastic coefficient matrices [38].

In general, the filters discussed above are UI-specific because of the significant differences between both the different types of UIs and the solutions designed to address them. However, in many practical applications, much more complicated UIs may be encountered. Recently, the minimum upper bound filter (MUBF) was proposed for a linear stochastic system corrupted by a generalized UI representing an arbitrary linear combination of dynamic UIs, random UIs, and deterministic UIs [39]. The result was further extended to a discrete-time non-linear stochastic system with a UI in its measurements, and an iterative optimization method for joint estimation and parameter identification was derived [40]. In multi-sensor bias estimation [41], the concept of a generalized sensor bias (GSB) has been proposed, which is represented by a dynamic model driven by a structured UI. By deriving an equivalent state-free measurement model and the corresponding UI-free dynamic model for the GSB, the LMMSE can be obtained via the orthogonal principle.

However, the application of the resultant filter is limited to the case in which all sensors have an identical measurement matrix, and only the GSB can be estimated. This problem motivates us to present and solve the problem of the joint estimation of the system state and the GSB under a UI in the case of GSB evolution.

This paper presents the joint estimation of the system state and GSB under a common UI in the case of bias evolution in a heterogeneous sensor network. To the best of the author’s knowledge, this is the first attempt to achieve this kind of the joint estimation. In detail, a two-stages cooperative strategy for pursuing consistent estimates in the distributed network is proposed. First, a joint UI-free evolution model is derived, along with its necessary and sufficient conditions for existence. In the derived model, the process noise and measurement noise are correlated. And the optimal local MMSE estimator with correlated noise is obtained. Second, based on the local estimates, the global estimation of the target state and UI are further achieved by network consensus. Through utilizing the global estimation of UI, the GSB is refined. The optimal estimates of the target state, GSB and UI are finally obtained.

Throughout the paper, the superscripts “-1”, “*T*” and “+” represent the inverse, transpose, and Moore-Penrose operations, respectively; “*I*” and “0” represent the identity matrix and the zero matrix, respectively, of the proper dimensions; “$0_{m\times n}$” represents the zero matrix with dimensions of m×n; diag$\left\{\cdot \right\}$ indicates a diagonal matrix; col$\left\{\cdot \right\}$ denotes column augmentation; E{·} denotes the operator of mathematical expectation; the superscript *i* indicates a matrix or variable related to the *i*th sensor; rank{·} denotes the rank of the specified matrix; the subscripts “*i*” and “*j*” indicate the *i*th and *j*th subblocks of a matrix, respectively; the superscripts “∧” and “”indicate the estimate and residual, respectively, of a random vector; the subscripts “k|k+1”represents the estimate of a vector at the time k using measurements up to time k + 1 and ⨂ denotes the Kronecker product.

## 2. Problem Formulation

Sensor bias (SB) is widespread in multi-sensor systems and originates from two types of sources: SBs of the first type are local SBs (LSBs), which are independent among sensors, such as calibration error, navigation bias and sensor faults/drift, whereas sensor biases of the second type are global SB (GLSB, also called UI in this work),which are common to all sensors, such as electronic countermeasures (ECMs) in target tracking. A GSB model has been proposed based on dynamic evolution driven by both independent LSBs and a common UI [41]. However, the corresponding filter has two shortcomings: first, all sensors must have an identical measurement matrix, and second, the system state and the UI cannot be estimated simultaneously because the SB estimation is obtained by deriving the equivalent GSB model neglecting system state and UI. Here, we present the two assumptions adopted in our work.

**Assumption 1.**  All measurements made by sensors in the network are continuously interrupted by the GSB, which includes LSBs and GLSB.

**Assumption 2.**  The network topology is fixed, and the target can be monitored by all sensors throughout the entire process. There is no fusion centre or lead sensor in this sensor network. In other words, all sensors are equal in status, and they reach a network consensus by exchanging information with their neighbours.

**Figure 1:**
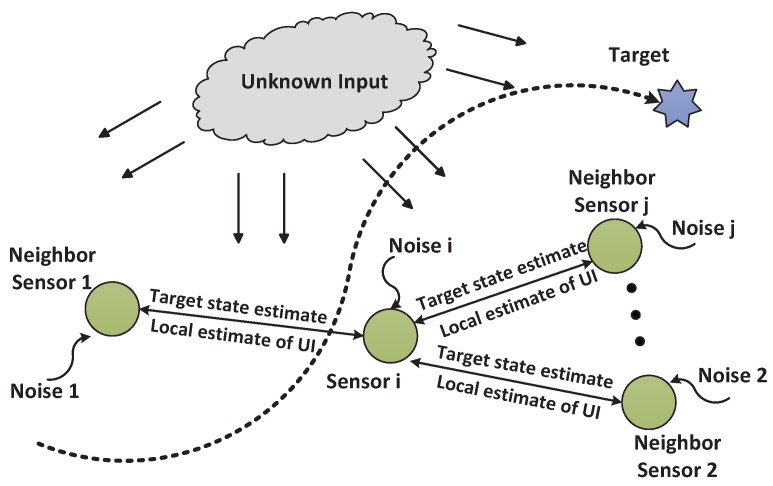
Collaborative target tracking in sensor network.

Consider a scenario of collaborative target tracking within a sensor network, as shown in Figure 1. A target is continuously moving within a surveillance zone. Each sensor node obtains raw measurements of the target corrupted by the GSB. At each step, each sensor obtains local estimates of the target state and GSB, exchanges its local system-state estimate with its neighbors, and obtains fused estimates of the target state and UI via network consensus. In other words, this sensor fusion process can be regarded as the joint distributed estimation of the target state, GSB and UI without the limitation that all sensor measurement matrices must be identical.

Consider a network of *s* sensor nodes. The network topology is represented by an undirected graph $G=\left(V,E,A\right)$, where $V=\left\{1,2,...,s\right\}$ denotes the set of nodes; E⊂V×V is the set of permissible communication links; and $A=\left[a_{ij}\right]$ is the graph topology matrix, the elements of which are defined as

$$a_{ij}=\left\{\begin{array}{c} 1,\;\left(i,j\right)\in E\;or\;i=j\\ 0,\;\left(i,j\right)\notin E \end{array}\right.$$

Let $N_{i}=\left\{i\in V:a_{ij}>0,i\neq j\right\}$ denote the set of neighbors of the *i*th sensor, and let $J_{i}=N_{i}\cup \left\{i\right\}$ denote the inclusive neighbor set of sensor *i*. Here, the presented network is assumed to be a connected graph. Otherwise, at least two separate sub-networks would exist, and hence, information could only be fused within each sub-network instead of throughout the entire network because information could not be exchanged between the two.

Here, we formulate the following problem of distributed sensor fusion in the presence of time-varying sensors subject to a common UI:

(1) $$\begin{array}{ccc} x_{k+1} & = & \Phi_{k}x_{k}+\zeta_{k}, \end{array}$$

(2) $$\begin{array}{ccc} y_{k+1}^{i} & = & H_{k+1}^{i}x_{k+1}+N_{k+1}^{i}b_{k+1}^{i}+v_{k+1}^{i} \end{array}$$

(3) $$\begin{array}{ccc} b_{k+1}^{i} & = & F_{k}^{i}b_{k}^{i}+G_{k}^{i}d_{k}+w_{k}^{i} \end{array}$$

where $x_{k}\in {ℜ}^{n}$ is the state vector; $y_{k}^{i}\in {ℜ}^{m_{i}}$ is the measurement vector of the *i*th sensor; $b_{k}^{i}\in {ℜ}^{p_{i}}$ is the *i*th GSB, where $i=\left\{1,2,...,s\right\}$; $d_{k}\in {ℜ}^{q}$ is the common UI; and $\zeta_{k}$, $w_{k}^{i}$ and $v_{k}^{i}$ are all independent, zero-mean, Gaussian, white-noise components with covariances of $Q_{k}$, $S_{k}^{i}$ and $R_{k}^{i}$, respectively. The initial target state $x_{0}$ and *i*th GSB state $b_{0}^{i}$ are Gaussian distributed with respective known means of ${\bar{x}}_{0}$ and ${\bar{b}}_{0}^{i}$ and associated covariances of $\Sigma_{0}^{x}$ and $\Sigma_{0}^{i,b}$. $\Phi_{k}$, $H_{k}^{i}$, $N_{k}^{i},$ $F_{k}^{i},$ and $G_{k}^{i}$ are known and have appropriate dimensions. Under the assumption that $m^{i}>p^{i}\geq q^{i}$, the $G_{k}^{i}$ are of full column rank.

Our aim is to design an unbiased minimum-variance filter to jointly estimate the target state $x_{k}$, the GSBs $b_{k}^{i}$ and the UI $d_{k}$ given the distributed measurements recorded up to time *k*.

**Remark 1.**  *The GSB $b_{k}^{i}$ in Equation (3) represents a generalized bias which includes multiple bias types as the special case:*

*(1).*  If $F_{k}^{i}=I$, $G_{k}^{i}=0$, $S_{k}^{i}=0$, then $b_{k+1}^{i}=b_{k}^{i}$ represents a constant-value bias.
*(2).*  If $F_{k}^{i}\neq 0$, $G_{k}^{i}=0$, then $b_{k+1}^{i}=F_{k}^{i}b_{k}^{i}+w_{k}^{i}$ represents a time-varying bias with known dynamic model.
*(3).*  If both $F_{k}^{i}=0$, $G_{k}^{i}=0$, then $b_{k+1}^{i}=w_{k}^{i}$ represents a zero-mean random error.
*(4).*  If $G_{k}^{i}\neq 0$, then $b_{k}^{i}$ represents the dynamically evolving bias driven by arbitrary UI.

The second type of the UI mentioned in the introduction is equivalent to the case (1).

The third type of the UI mentioned in the introduction is equivalent to the case (4).

The first and fourth types of the UI mentioned in the introduction can be contributed to the case (4) when these two types of UI can be decoupled from Equation (3).

*The above four types of UI can be accommodated within the single-model system proposed in this work. However, for the fifth type of UI mentioned in the introduction, it is usually regarded as a randomly switching parameter obeying a known Markov chain which is classified to the multiple-models system, so this type of UI cannot be accommodated within the presented model in this work. Nevertheless, for this type of UI, if GSB model can be established for the each mode of Markov chain. According to Equation (3), GSB model can be written as*

(4) $$b_{k+1}^{i,j}=F_{k}^{i,j}b_{k}^{i,j}+G_{k}^{i,j}d_{k}+w_{k}^{i,j},j=1,2...r$$

*where r represents the number of states of the Markov chain. Meanwhile, according to Equation (10), the decoupling condition of the corresponding GSB model can be also confirmed as*

(5) $$rank\left({\bar{H}}_{k+1}^{i}{\bar{G}}_{k}^{i,j}\right)=rank\left({\bar{G}}_{k}^{i,j}\right),j=1,2...r$$

These decoupling conditions for a UI obeying a Markov chain are more stringent than those considered in this work, as Equation (5) must hold for every r. Then the fifth type of UI can be accommodated within the proposed framework.

**Remark 2.**  There are two distinct strategies of networked sensor fusion. The first is the centralized strategy [41,42], i.e., all sensors transmit their measurements to a fusion centre, which is responsible for integrated data processing for state estimation and/or parameter identification. The second is the distributed strategy [25], i.e., each sensor processes its own measurements and then shares its processing results with its neighbors in an iterative manner to achieve consistent fusion. Obviously, the distributed fusion strategy allows the computational burden to be shared among sensors and consequently is more desirable for large-scale networks. However, distributed fusion is much more complex, involving both local processing and global fusion. Currently, distributed fusion in the presence of time-varying sensor biases driven by a common UI remains an open problem.

**Remark 3.**  Concerning our proposed problem, there exist two possible solution approaches. One is iterative optimization between state estimation and UI identification, as in the well-known expectation-maximization (EM) scheme [25]. As shown later via simulation, the EM scheme is not desirable here because it must treat the UI as a constant or slowly varying parameter in each iterative window. The other approach is UI decoupling plus state estimation, which serves as the basis for our proposed method, as derived later. The main technical difficulty of this approach is to determine how to adaptively decouple the UI and then fuse the local state and UI estimates.

## 3. Main Results

Because of the presence of the UI, the target state and GSB cannot be estimated directly. In this section, an equivalent UI-free dynamic model of the bias is derived and the necessary and sufficient conditions for the UI-free model are explored. Afterwards, optimal estimators, in the sense of minimum variance, are proposed for the target state $x_{k}$ and the local GSBs $b_{k}^{i}$.

### 3.1. Local LMMSE Filter for System State and Local SB

**Lemma 1.**  *For matrices* Γ, Ψ *and I with proper dimensions, if the matrix* Ψ *is of full column rank, the equality*

(6) $$\Psi \left[I-{\left(\Gamma \Psi \right)}^{+}\left(\Gamma \Psi \right)\right]=0$$

*will hold if and only if*

(7) rank{ΓΨ}=rank{Ψ}

**Proof.**  See Appendix A. ☐

For convenience of derivation, the model defined in Equations (1) and (2) can be rewritten as follows:

(8) $$\begin{array}{ccc} z_{k+1}^{i} & = & A_{k}^{i}z_{k}^{i}+{\bar{G}}_{k}^{i}d_{k}+{\bar{\zeta }}_{k}^{i} \end{array}$$

(9) $$\begin{array}{ccc} y_{k+1}^{i} & = & {\bar{H}}_{k+1}^{i}z_{k+1}^{i}+v_{k+1}^{i} \end{array}$$

where $z_{k}^{i}=\mathrm{col}\left\{x_{k},b_{k}^{i}\right\}$, $A_{k}^{i}=\mathrm{diag}\left\{\Phi_{k},F_{k}^{i}\right\}$, ${\bar{G}}_{k}^{i}=\mathrm{col}\left\{0_{n\times q},G_{k}^{i}\right\}$, ${\bar{\zeta }}_{k}^{i}=\mathrm{col}\left\{\zeta_{k},w_{k}^{i}\right\}$ and ${\bar{H}}_{k}^{i}=\left[\begin{array}{cc} H_{k}^{i} & N_{k}^{i} \end{array}\right]$. It is easy to find that ${\bar{S}}_{k}^{i}≐$E$\left[{\bar{\zeta }}_{k}^{i}{\left({\bar{\zeta }}_{k}^{i}\right)}^{T}\right]=$diag$\left\{Q_{k},S_{k}^{i}\right\}$.

**Theorem 1.**  *(UI decoupling). Consider the system model defined in Equations (8) and (9). If and only if*

(10) $$rank\left({\bar{H}}_{k+1}^{i}{\bar{G}}_{k}^{i}\right)=rank\left({\bar{G}}_{k}^{i}\right)$$

*then the UI will be decoupled from $z_{k+1}^{i}$, i.e., there will exist the following UI-free dynamic model of the sensor bias:*

(11) $$z_{k+1}^{i}={\bar{F}}_{k}^{i}A_{k}^{i}z_{k}^{i}+{\bar{C}}_{k}^{i}y_{k+1}^{i}+{\bar{B}}_{k}^{i}{\bar{w}}_{k}^{i}$$

*where*

(12) $$\begin{array}{ccc} \Pi_{k}^{i} & = & {\left({\bar{H}}_{k+1}^{i}{\bar{G}}_{k}^{i}\right)}^{+} \end{array}$$

(13) $$\begin{array}{ccc} {\bar{C}}_{k}^{i} & = & {\bar{G}}_{k}^{i}\Pi_{k}^{i} \end{array}$$

(14) $$\begin{array}{ccc} {\bar{F}}_{k}^{i} & = & I_{n+p^{i}}-{\bar{C}}_{k}^{i}{\bar{H}}_{k+1}^{i} \end{array}$$

(15) $$\begin{array}{ccc} {\bar{w}}_{k}^{i} & = & col\{{\bar{\zeta }}_{k}^{i},v_{k+1}^{i}\} \end{array}$$

(16) $$\begin{array}{ccc} {\bar{B}}_{k}^{i} & = & \left[\begin{array}{cc} {\bar{F}}_{k}^{i} & -{\bar{C}}_{k}^{i} \end{array}\right] \end{array}$$

**Proof.**  See Appendix B. ☐

**Remark 4.**  *If the matrices* Γ *and* Ψ *satisfy Equation (7), then the row rank of the matrix* Γ *should be no less than the column rank of the matrix* Ψ *in Lemma 1. As shown in Equation (10), this means that the maximum number of UIs that can be decoupled from the system must be fewer than the number of independent measurement components of all sensors.*

As shown in Equation (11), the measurements $y_{k}^{i}$ are introduced into the equivalent bias dynamic model to decouple $d_{k}$ from $b_{k}^{i}.$ Defining ${\bar{Q}}_{k}^{i}≐$E$\left({\bar{w}}_{k}^{i}{\left({\bar{w}}_{k}^{i}\right)}^{T}\right)$ and $M_{k+1}^{i}≐$E$\left({\bar{B}}_{k}^{i}{\bar{w}}_{k}^{i}{\left(v_{k+1}^{i}\right)}^{T}\right)$, we have

(17) $$\begin{array}{ccc} {\bar{Q}}_{k}^{i} & = & \mathrm{diag}\{{\bar{S}}_{k}^{i},R_{k+1}^{i}\} \end{array}$$

(18) $$\begin{array}{ccc} M_{k+1}^{i} & = & -{\bar{C}}_{k}^{i}R_{k+1}^{i} \end{array}$$

Here, $M_{k+1}^{i}\neq 0$ means that the process noise ${\bar{w}}_{k}^{i}$ is correlated with the measurement noise $v_{k}^{i}$. Thus, designing an unbiased minimum-variance linear filter for the system defined in Equations (1) and (2) is equivalent to finding the MMSE estimation for the system defined in Equations (9) and (11) with correlated process noise and measurement noise.

**Theorem 2.**  *The MMSE estimators for the UI-free system defined in Equations (9) and (11) have the following recursion relations:*

(19) $$\begin{array}{ccc} {\hat{z}}_{k+1|k}^{i} & = & {\bar{F}}_{k}^{i}A_{k}^{i}{\hat{z}}_{k|k}^{i}+{\bar{C}}_{k}^{i}y_{k+1}^{i} \end{array}$$

(20) $$\begin{array}{ccc} P_{k+1|k}^{i} & = & {\bar{F}}_{k}^{i}A_{k}^{i}P_{k|k}^{i}{\left({\bar{F}}_{k}^{i}A_{k}^{i}\right)}^{T}+{\bar{B}}_{k}{\bar{Q}}_{k}{\left({\bar{B}}_{k}^{i}\right)}^{T}+{\bar{C}}_{k}^{i}R_{k+1}{\left({\bar{C}}_{k}^{i}\right)}^{T} \end{array}$$

(21) $$\begin{array}{ccc} {\hat{z}}_{k+1|k+1}^{i} & = & {\hat{z}}_{k+1|k}^{i}+K_{k+1}^{i}\left[y_{k+1}^{i}-{\bar{H}}_{k+1}^{i}{\hat{z}}_{k+1|k}^{i}\right] \end{array}$$

(22) $$\begin{array}{ccc} P_{k+1|k+1}^{i} & = & P_{k+1|k}^{i}-K_{k+1}^{i}\left[{\bar{H}}_{k+1}^{i}P_{k+1|k}^{i}+{\left(M_{k+1}^{i}\right)}^{T}\right] \end{array}$$

*where the estimator gain is*

(23) $$K_{k+1}^{i}=\left[P_{k+1|k}^{i}{\left({\bar{H}}_{k+1}^{i}\right)}^{T}+M_{k+1}^{i}\right]{\left[{\bar{H}}_{k+1}^{i}P_{k+1|k}^{i}{\left({\bar{H}}_{k+1}^{i}\right)}^{T}+{\bar{H}}_{k+1}^{i}M_{k+1}^{i}+{\left({\bar{H}}_{k+1}^{i}M_{k+1}^{i}\right)}^{T}+R_{k+1}^{T}\right]}^{-1}$$

**Proof.**  The term ${\bar{C}}_{k}^{i}y_{k+1}^{i}$ is an additive known input. Equations (9) and (11) are linear, with additional Gaussian noise. Hence, the optimal filter with correlated process noise and measurement noise [43] can be directly utilized. ☐

**Figure 2:**
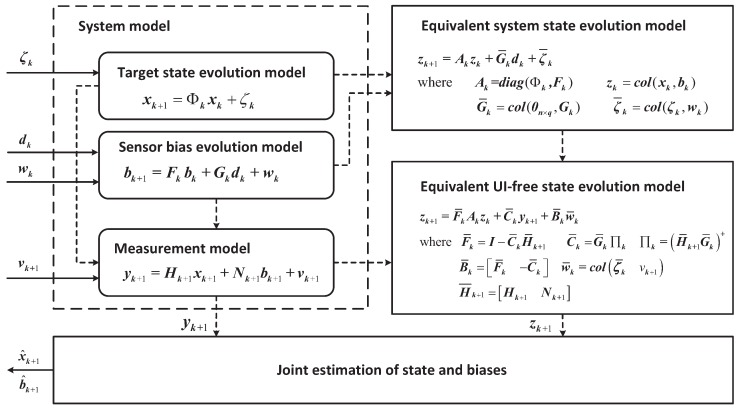
The scheme of joint MMSE estimation of the state and the SB.

The framework and pseudo-code for the proposed local MMSE estimation are presented in Figure 2 and Table 1, respectively.

### 3.2. Consensus Estimation of the Target State and the UI

Given the local estimate ${\hat{z}}_{k+1|k+1}^{i}$ of the *i*th sensor derived in the previous section, each sensor exchanges its own estimates with its neighbours to reach consistent consensus estimates of $x_{k}$ and $d_{k}$. The corresponding consensus estimates consist of two parts: an average consensus filter for $x_{k}$ and a distributed least-squares (DLS) estimate for $d_{k}$.

#### 3.2.1. Local LS Estimator of UI

Let us rewrite the sensor bias evolution model defined in Equation (3) as

(24) $$\begin{array}{c} \begin{array}{c} G_{k}^{i}d_{k}=b_{k+1}^{i}-F_{k}^{i}b_{k}^{i}-w_{k}^{i}\\ \;\;\;\;\;\;\;\;\;={\hat{b}}_{k+1|k+1}^{i}-F_{k}^{i}{\hat{b}}_{k|k}^{i}+\left({\tilde{b}}_{k+1|k+1}^{i}-F_{k}^{i}{\tilde{b}}_{k|k}^{i}-w_{k}^{i}\right)\\ \;\;\;\;\;\;\;\;\;={\bar{b}}_{k}^{i}+\xi_{k}^{i} \end{array} \end{array}$$

where ${\bar{b}}_{k}^{i}={\hat{b}}_{k+1|k+1}^{i}-F_{k}^{i}{\hat{b}}_{k|k}^{i}$ and $\xi_{k}^{i}={\tilde{b}}_{k+1|k+1}^{i}-F_{k}^{i}{\tilde{b}}_{k|k}^{i}-w_{k}^{i}$. ${\hat{z}}_{k+1|k+1}^{i}$ is the unbiased estimate of $z_{k+1}^{i}$ in Section 2. It is easily confirmed that $E\left(\xi_{k}^{i}\right)=0$. We can rewrite Equation (24) as the following compact expression:

$$G_{k}d_{k}=B_{k}+\xi_{k}$$

where $G_{k}=\mathrm{col}\left\{G_{k}^{j}:j\in J_{i}\right\}$, $B_{k}=\mathrm{col}\left\{{\bar{b}}_{k}^{j}:j\in J_{i}\right\}$, and $\xi_{k}=\mathrm{col}\left\{\xi_{k}^{j}:j\in J_{i}\right\}$ Furthermore, based on the local GSB estimates of the *i*th node and its neighbours, the local DLS estimate of $d_{k}$ and the corresponding covariance of the *i*th sensor are obtained as follows:

(25) $$\begin{array}{ccc} {\hat{d}}_{k}^{i} & = & G_{k}^{T}{\left(G_{k}\right)}^{-1}G_{k}^{T}B_{k} \end{array}$$

(26) $$\begin{array}{ccc} P_{k}^{dd,i} & = & {\left(G_{k}^{T}G_{k}\right)}^{-1} \end{array}$$

#### 3.2.2. Average Consensus Filter for the Target State and the UI

Now, estimates of the target state and the UI have been obtained, but they are local. In other words, the estimates from different nodes are not identical to each other. Therefore, an average consensus fusion (ACF) is presented here to obtain a uniform result at each node via iterative interaction.

Let $W\left(l\right)=\left[w_{ij}\left(l\right)\right]$ denote the linear weight matrix in the *l*th step of iteration. Here, $w_{ij}\left(l\right)$ is the measurement weight of node *j* at node *i*, which satisfies

$$\left\{\begin{array}{c} \sum_{j=1}^{s}w_{ij}\left(l\right)=1\\ \sum_{i=1}^{s}w_{ij}\left(l\right)=1\\ w_{ij}\left(l\right)\geq 0 \end{array}\right.$$

Let $W_{k}^{x}\left(l\right)=\left[w_{ij}^{x}\left(l\right)\right]$ and$\;W_{k}^{d}\left(l\right)=\left[w_{ij}^{d}\left(l\right)\right]$ represent the weight matrices for the target state and the UI, respectively.

Given the consensus covariances of the state estimate and bias estimate of node *i* after the *l*th iteration, denoted by $P_{k|k}^{xx,i}\left(l\right)$ and $P_{k}^{dd,i}\left(l\right)$, respectively, we design the weight matrices as follows:

$$\left\{\begin{array}{c} w_{ij}^{x}\left(l+1\right)=\frac{tr\left[{\left(P_{k|k}^{xx,j}\left(l\right)\right)}^{-1}\right]}{\sum_{t\in J_{i}}tr\left[{\left(P_{k|k}^{xx,t}\left(l\right)\right)}^{-1}\right]}\;j\in J_{i}\\ w_{ij}^{x}\left(l+1\right)=0\;\;\;\;otherwise \end{array}\right.$$

and

$$\left\{\begin{array}{c} w_{ij}^{d}\left(l+1\right)=\frac{tr\left[{\left(P_{k}^{dd,j}\left(l\right)\right)}^{-1}\right]}{\sum_{t\in J_{i}}tr\left[{\left(P_{k}^{dd,t}\left(l\right)\right)}^{-1}\right]}\;j\in J_{i}\\ w_{ij}^{d}\left(l+1\right)=0\;\;\;\;otherwise \end{array}\right.$$

such that local estimates of higher accuracy will be assigned higher weights in the weighted fusion process.

Then, the initial values can be set to ${\hat{x}}_{k|k}^{i}\left(0\right)≐{\hat{x}}_{k|k}^{i}$, ${\hat{d}}_{k}^{i}\left(0\right)≐{\hat{d}}_{k}^{i}$, $P_{k|k}^{xx,i}\left(0\right)≐P_{k|k}^{xx,i}$, and $P_{k}^{dd,i}\left(0\right)≐P_{k}^{dd,i}$, where ${\hat{x}}_{k|k}^{i}$ and $P_{k|k}^{xx,i}$ are subblocks of ${\hat{z}}_{k|k}^{i}$ and $P_{k|k}^{i}$, respectively, i.e., ${\hat{z}}_{k|k}^{i}=\left[\begin{array}{c} {\hat{x}}_{k|k}^{i}\\ {\hat{b}}_{k|k}^{i} \end{array}\right]$ and $P_{k|k}^{i}=\left[\begin{array}{cc} P_{k|k}^{xx,i} & P_{k|k}^{xb,i}\\ P_{k|k}^{bx,i} & P_{k|k}^{bb,i} \end{array}\right]$.

**Theorem 3.**  *Consider the multi-sensor system defined in Equations (1)–(3), with topology $G=\left(V,E,A\right)$ and weight matrices $\left[w_{ij}^{x}\left(l\right)\right]$ and $\left[w_{ij}^{d}\left(l\right)\right]$ for the target state and UI, respectively, at the lth step of iteration. The ACFs for $x_{k}$ and $d_{k}$ are*

(27) $$\begin{array}{ccc} {\hat{x}}_{k|k}^{i}\left(l+1\right) & = & \sum_{j\in J_{i}}w_{ij}^{x}\left(l\right){\hat{x}}_{k|k}^{j}\left(l\right) \end{array}$$

(28) $$\begin{array}{ccc} {\hat{d}}_{k}^{i}\left(l+1\right) & = & \sum_{j\in J_{i}}w_{ij}^{x}\left(l\right){\hat{d}}_{k}^{j}\left(l\right) \end{array}$$

*where*

(29) $$\begin{array}{ccc} P_{k|k}^{xx,i}\left(l+1\right) & = & \sum_{j\in J_{i}}w_{ij}^{x}\left(l\right)\left[P_{k|k}^{xx,j}\left(l\right)+{\hat{x}}_{k|k}^{j}\left(l\right){\left({\hat{x}}_{k|k}^{j}\left(l\right)\right)}^{T}\right]-{\hat{x}}_{k|k}^{i}\left(l+1\right){\left({\hat{x}}_{k|k}^{i}\left(l+1\right)\right)}^{T} \end{array}$$

(30) $$\begin{array}{ccc} P_{k}^{dd,i}\left(l+1\right) & = & \sum_{j\in J_{i}}w_{ij}^{d}\left(l\right)\left[p_{k}^{dd,j}\left(l\right)+{\hat{d}}_{k}^{j}\left(l\right){\left({\hat{d}}_{k}^{j}\left(l\right)\right)}^{T}\right]-{\hat{d}}_{k}^{i}\left(l+1\right){\left({\hat{d}}_{k}^{i}\left(l+1\right)\right)}^{T} \end{array}$$

**Proof.**  See Appendix C. ☐

In the *i*th node, Equations (27)–(30) will remain iterative until

(31) $$\frac{\left|tr\left[P_{k|k}^{xx,i}\left(l+1\right)\right]-tr\left[P_{k|k}^{xx,i}\left(l\right)\right]\right|}{tr\left[P_{k|k}^{xx,i}\left(l\right)\right]}\leq \eta^{x}$$

and

(32) $$\frac{\left|tr\left[P_{k}^{dd,i}\left(l+1\right)\right]-tr\left[P_{k}^{dd,i}\left(l\right)\right]\right|}{tr\left[P_{k}^{dd,i}\left(l\right)\right]}\leq \eta^{d}$$

where $\eta^{x}$ and $\eta^{d}$ are positive thresholds for system state and UI, respectively.

#### 3.2.3. Refinement of the Local Bias Estimates

After the consensus iteration of ${\hat{x}}_{k|k}^{i}$ and ${\hat{d}}_{k-1}^{i}$, the sensor biases can be refined according to Equation (3), that is,

(33) $${\hat{b}}_{k|k}^{i}=F_{k-1}^{i}{\hat{b}}_{k-1|k-1}^{i}+G_{k-1}^{i}{\hat{d}}_{k-1}^{i}$$

where ${\hat{d}}_{k-1}^{i}$ is the consensus estimate at the kth sampling instant. The covariance of ${\hat{b}}_{k|k}^{i}$ is as follows:

(34) $$P_{k|k}^{bb,i}=F_{k-1}^{i}P_{k-1|k-1}^{bb,i}{\left(F_{k-1}^{i}\right)}^{T}+G_{k-1}^{i}P_{k-1}^{dd,i}{\left(G_{k-1}^{i}\right)}^{T}+S_{k-1}^{i}$$

Thus, the estimates ${\hat{b}}_{k|k}^{i}$, $P_{k|k}^{bb,i}$, ${\hat{x}}_{k|k}^{i}$, $P_{k|k}^{xx,i}$, ${\hat{d}}_{k-1}^{i}$ and $P_{k-1}^{dd,i}$ will be output as the final results at the kth sampling instant.

**Figure 3:**
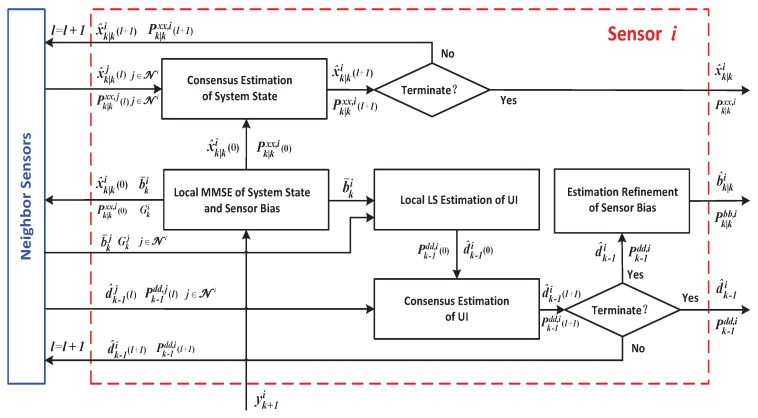
The scheme of the consensus estimation process for the target state and the UI.

The framework and pseudo-code for the iterative consensus process are presented in Figure 3 and Table 2, respectively.

**Remark 5.**  In this paper, a fusion algorithm for multiple cooperative sensors subject to interference from GSBs is proposed. However, when this algorithm is applied to continuous target tracking in a wireless sensor networks (WSN), a new problem should be considered. Because of the sensors’ limited detection capabilities and low energy storage, a WSN is divided into many clusters. Each cluster forms a sub-WSN and is responsible for tracking the target for a certain period of time. When the target moves out of the surveillance area of the current cluster, one or more new clusters must be generated dynamically; thus, a strategy for generating such clusters must be proposed. This strategy must specify how to choose the leader and followers in each cluster and how to transmit estimate information between clusters. This problem will be an interesting topic for future research regarding the joint optimization of signal processing and distributed cooperative detection. Currently, this topic has been tried in our research group [44].

**Remark 6.**  For the case of multiple-target tracking with dense clutter, the association relationships between the targets and measurements are unknown. Therefore, the problem of data association must be considered when our proposed algorithm is applied for multiple-target tracking. To this end, the optimal estimation for a single target must be extended to the joint optimization of state estimation and target identification for multiple targets. However, because of the limited detection capabilities of nodes in a WSN, as also mentioned in Remark 5, a WSN is often divided into many clusters, each of which monitors only a relatively small region. Consequently, the probability of multiple targets being present in any single cluster is statistically low. Therefore, our algorithm is focused only on single-target tracking.

## 4. Numerical Example

Consider examples of distributed networks tracking one constant-velocity target. The target state is ${\left[x_{k},{\dot{x}}_{k},y_{k},{\dot{y}}_{k}\right]}^{T}$, where ${\left[x_{k},{\dot{x}}_{k}\right]}^{T}$ and ${\left[y_{k},{\dot{y}}_{k}\right]}^{T}$ represent the target’s Cartesian position and velocity on the x and y axes, respectively. The sampling interval is T=1s, and the number of the samples is 60. Each sensor node synchronously measures the position of the target at each sampling instant. The measurements of each sensor node are corrupted by a local dynamic system bias, and all local system biases originate from a common UI. This situation can be modelled using Equations (1) and (3) with the following parameters:

$$\Phi_{k}=I_{2}⊗\left[\begin{array}{cc} 1 & T\\ 0 & 1 \end{array}\right],F_{b}^{i}=\left[\begin{array}{cc} -0.05 & -0.84\\ 0.5170 & 0.8069 \end{array}\right],G_{k}^{i}=\left[\begin{array}{c} 2.58\\ -2.5008 \end{array}\right],H_{k}^{i}=\left[\begin{array}{cccc} 1 & 0 & 0 & 0\\ 0 & 0 & 1 & 0 \end{array}\right]$$

The initial position of the target is ${\left[0,0\right]}^{T}$, in units of m, and the initial velocity is ${\left[50,60\right]}^{T}$, in m/s. The initial value of the bias is ${\bar{b}}_{0}^{i}={\left[1,1\right]}^{T}$, in m. The covariance matrices are as follows (all in m${}^{2}$/s${}^{4}$):

$$R_{k}^{i}=\left[\begin{array}{cc} 16 & 0\\ 0 & 16 \end{array}\right],Q_{k}=0.01\times I_{2}⊗\left[\begin{array}{c} T^{2}/2\\ T \end{array}\right]\times \left[\begin{array}{cc} T^{2}/2 & T \end{array}\right],S_{k}^{i}=\left[\begin{array}{cc} 0.36 & 0.342\\ 0.342 & 0.3249 \end{array}\right]$$

### 4.1. A Distributed Network of Twelve Sensors

Consider an example of a distributed network of twelve sensors, and the matrices $N_{k}^{i}$ for the 12 sensor nodes are

$$\begin{array}{ccc} N_{k}^{1} & = & diag\left(3,-5\right),\;N_{k}^{2}=diag\left(7,1\right),\;\;\;N_{k}^{3}=diag\left(-3,7\right),\;\;N_{k}^{4}=diag\left(4,-6\right),\\ N_{k}^{5} & = & diag\left(7,3\right),\;\;\;N_{k}^{6}=diag\left(2,-8\right),\;\;N_{k}^{7}=diag\left(-7,5\right),\;\;N_{k}^{8}=diag\left(3,8\right),\\ N_{k}^{9} & = & diag\left(2,-5\right),\;\;N_{k}^{10}=diag\left(7,4\right),\;\;N_{k}^{11}=diag\left(-9,8\right),\;\;N_{k}^{12}=diag\left(8,4\right) \end{array}$$

**Figure 4:**
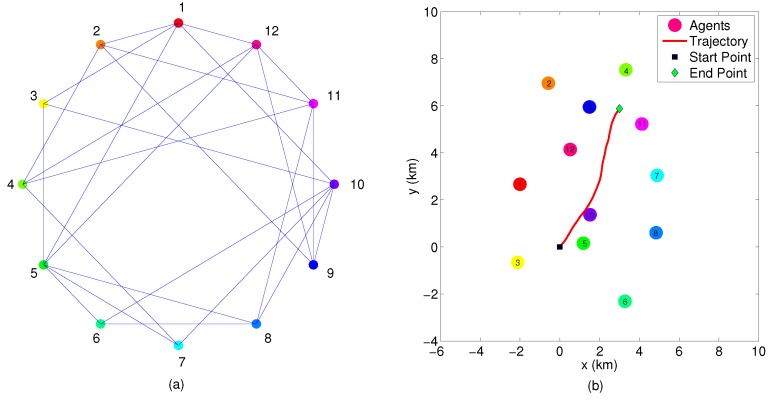
Examples of networks with 12 sensor nodes: (**a**) Graph-topological map of the network (**b**) Sensors’ real locations and target trajectory.

The dimensions of the surveillance area are 16 km × 14 km. The topological map is depicted in Figure 4a, and the sensors’ true locations and the target trajectory are depicted in Figure 4b.

**Figure 5:**
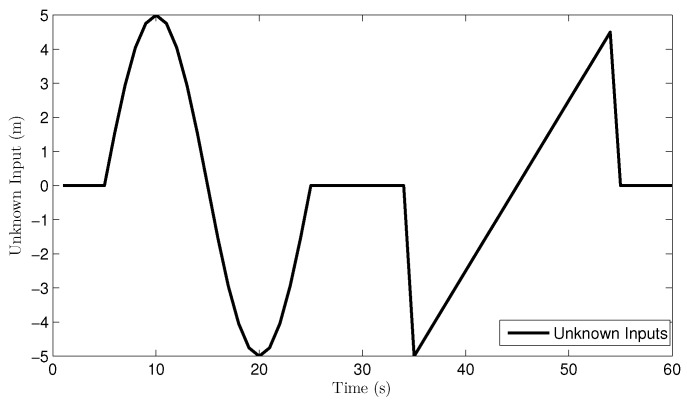
Real unknown input in the multi-sensor system.

The UI, plotted in Figure 5, is

$$d_{k}=\left\{\begin{array}{c} 0m\;\;\;\;0<t\leq 5\\ 5\times \sin\frac{\pi \left(t-5\right)}{10}m\;\;5<t\leq 25\\ 0m\;\;\;\;25<t\leq 35\\ 0.5\times \left(t-45\right)m\;\;35<t\leq 55\\ 0m\;\;\;\;55<t\leq 60 \end{array}\right.$$

**Figure 6:**
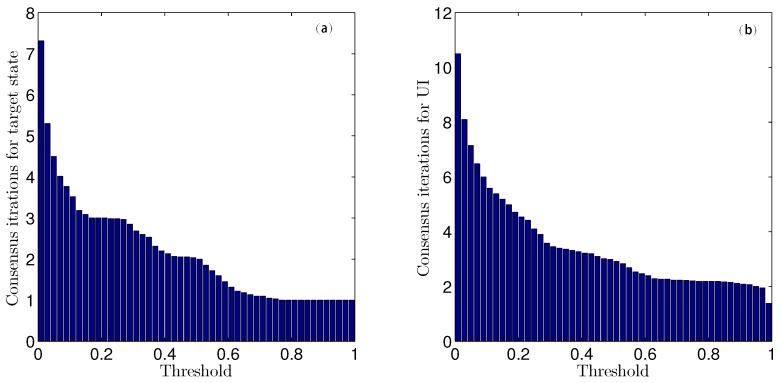
The mean numbers of iteration steps for (**a**) state and (**b**) UI estimation with different thresholds.

The histograms shown in Figure 6 depict the numbers of steps of iteration for different thresholds. Clearly, the number of iterations required for consensus increases with a decreasing threshold. Moreover, when the threshold is greater than 0.1, the number of iteration steps varies only slowly. However, when the threshold is less than 0.1, the number of iteration steps increases sharply, and the computational cost is dramatically increased. Considering both calculation and accuracy, the consensus threshold values for both target state and UI estimation are chosen to be $\eta^{x}=\eta^{d}=$ 0.1.

In accordance with Theorem 2, the rank of the filter matrix is *rank*$\left({\bar{H}}_{k+1}^{i}{\bar{G}}_{k}^{i}\right)=$*rank*$\left({\bar{G}}_{k}^{i}\right)=1$, which satisfies Equation (10).

The proposed algorithm is compared with the EM algorithm [25] in this simulation. The iteration threshold for the EM algorithm is $\delta =10^{-4}$, and the processing window length is l=5. We also apply the standard Kalman filter, neglecting the presence of a UI. The estimation of the Kalman filter shows that the fusion performance will deteriorate considerably when bias exists in the sensor measurements.

**Figure 7:**
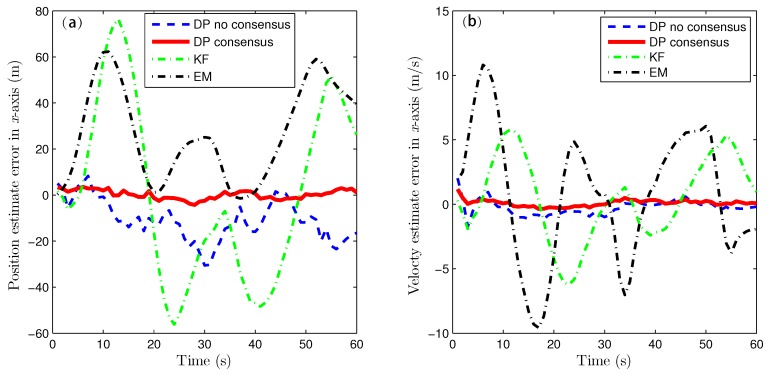
State estimation errors in the x direction using the KF, DP consensus, DP no consensus and EM methods for sensor 4. (**a**) position estimation; (**b**) velocty estimation.

**Figure 8:**
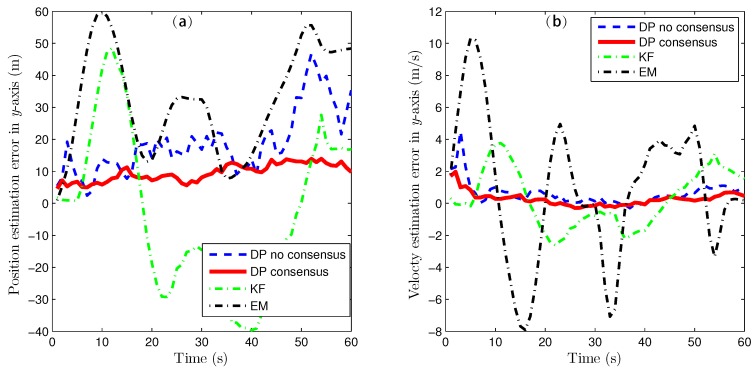
State estimation errors in the y direction using the KF, DP consensus, DP no consensus and EM methods for sensor 4. (**a**) position estimation; (**b**) velocty estimation.

**Figure 9:**
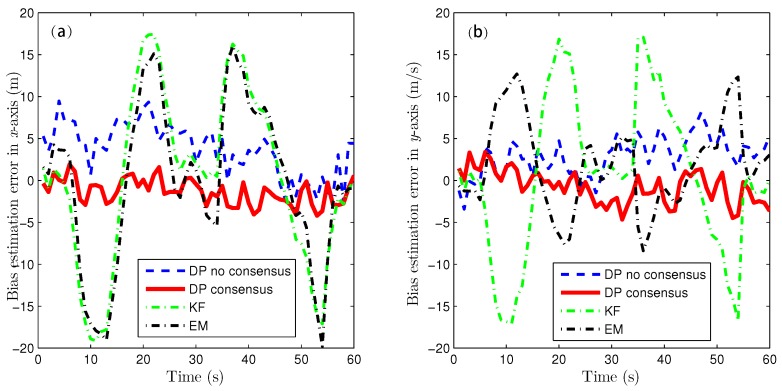
Bias estimation errors using the KF, DP consensus, DP no consensus and EM methods for sensor 4. (**a**) x-axis; (**b**) y-axis.

Because of the page limitation and the similarity in the data variation trends among the simulated sensors, only the estimation results for the fourth sensor are presented here. Figure 7 and Figure 8 show the target-state estimation errors for the Kalman filter (called ‘KF’), the EM method, and the proposed method without consensus (called ‘DP no consensus’) and with consensus (called ‘DP consensus’). It can be seen that in the DP methods, the UI is decoupled from the system. Furthermore, the estimation error of the DP consensus method is less than that of the DP no consensus method, and the estimation accuracy is also improved for the DP consensus method. It is also observed that the estimation results based on the KF and EM methods are not as good as those achieved using the DP methods. Two reasons for these findings can be identified. One is that the UI is ignored in the KF method, causing the KF algorithm to become invalid. The other is that the UI is assumed to take a constant value in the iterative process of the EM method. Similar conclusions can also be drawn from the results of the sensor bias estimation, as shown in Figure 9.

**Figure 10:**
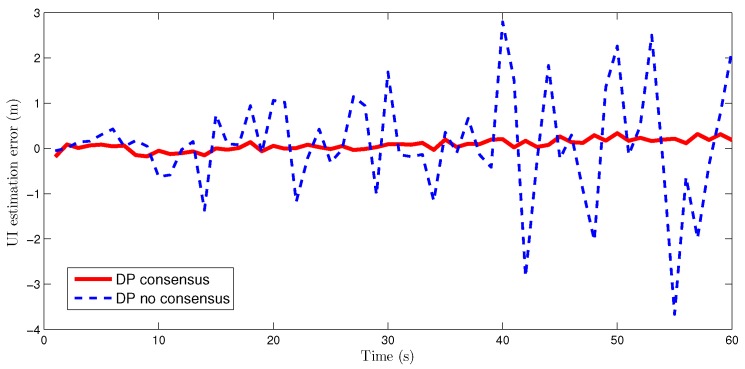
UI estimation errors of the DP consensus and DP no consensus methods for sensor 4.

The estimation errors for the UI based on the DP methods with and without consensus are plotted in Figure 10. It can be seen that the UI estimation error is greatly reduced by the consensus processing.

The mean and peak values of the estimation errors for the target state ${\left[x,\dot{x},y,\dot{y}\right]}^{T}$ and the bias ${\left[b_{x},b_{y}\right]}^{T}$ based on the KF, EM and DP methods are listed in Table 3 and Table 4, respectively. It can be seen from Table 3 that the mean estimation error values of the DP consensus method are all smaller than those of the KF, EM and DP no consensus methods. As shown in Table 4, the peak estimation error values are also smallest for the DP consensus must. Thus, the proposed method is proven to be effective.

The run times are also compared as listed in Table 5. It can be seen that the run time for the DP no consensus method is merely 0.33 s because of its non-iteration operation. After iteration, the run time of the DP consensus method is 4.89 s, which is nevertheless shorter than the 6.57 s required for the EM method.

### 4.2. A Distributed Network of Sixty Sensors

Now we consider an example of distributed network of sixty sensors, and the matrices $N_{k}^{i}$ of the sensor nodes are

$$\begin{array}{c} \begin{array}{cc} & N_{k}^{1}=N_{k}^{13}=N_{k}^{25}=N_{k}^{37}=N_{k}^{49}=diag\left(3,-5\right),N_{k}^{2}=N_{k}^{14}=N_{k}^{26}=N_{k}^{38}=N_{k}^{50}=diag\left(7,1\right),\\ & N_{k}^{3}=N_{k}^{15}=N_{k}^{27}=N_{k}^{39}=N_{k}^{51}=diag\left(-3,7\right),N_{k}^{4}=N_{k}^{16}=N_{k}^{28}=N_{k}^{40}=N_{k}^{52}=diag\left(4,-6\right),\\ & N_{k}^{5}=N_{k}^{17}=N_{k}^{29}=N_{k}^{41}=N_{k}^{53}=diag\left(7,3\right),N_{k}^{6}=N_{k}^{18}=N_{k}^{30}=N_{k}^{42}=N_{k}^{54}=diag\left(2,-8\right),\\ & N_{k}^{7}=N_{k}^{19}=N_{k}^{31}=N_{k}^{43}=N_{k}^{55}=diag\left(-7,5\right),N_{k}^{8}=N_{k}^{20}=N_{k}^{32}=N_{k}^{44}=N_{k}^{56}=diag\left(3,8\right),\\ & N_{k}^{9}=N_{k}^{21}=N_{k}^{33}=N_{k}^{45}=N_{k}^{57}=diag\left(2,-5\right),N_{k}^{10}=N_{k}^{22}=N_{k}^{34}=N_{k}^{46}=N_{k}^{58}=diag\left(7,4\right),\\ & N_{k}^{11}=N_{k}^{23}=N_{k}^{35}=N_{k}^{47}=N_{k}^{59}=diag\left(-9,8\right),N_{k}^{12}=N_{k}^{24}=N_{k}^{36}=N_{k}^{48}=N_{k}^{60}=diag\left(8,4\right) \end{array} \end{array}$$

**Figure 11:**
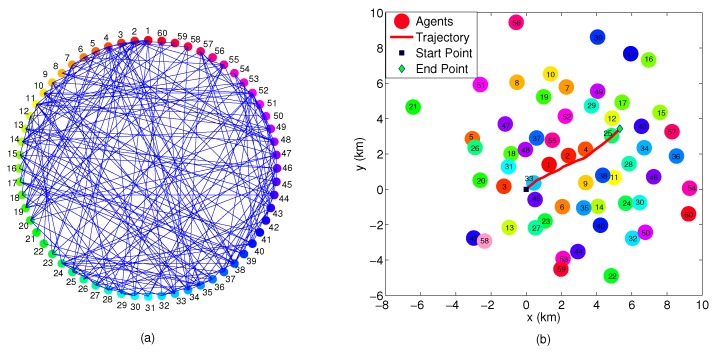
Examples of networks with 60 sensors: (**a**) Graph-topological map of the network; (**b**) Sensors’ true locations and target trajectory.

The dimensions of the surveillance area are 18 km × 16 km. The topological map is shown in Figure 11a. Sensors’ true locations and the target trajectory are depicted in Figure 11b.

In accordance with Theorem 2, the rank of filter matrix rank$\left({\bar{H}}_{k+1}^{i}{\bar{G}}_{k}^{i}\right)=$rank$\left({\bar{G}}_{k}^{i}\right)=1$ for each sensor in the network, which satisfies Equation (10).

**Figure 12:**
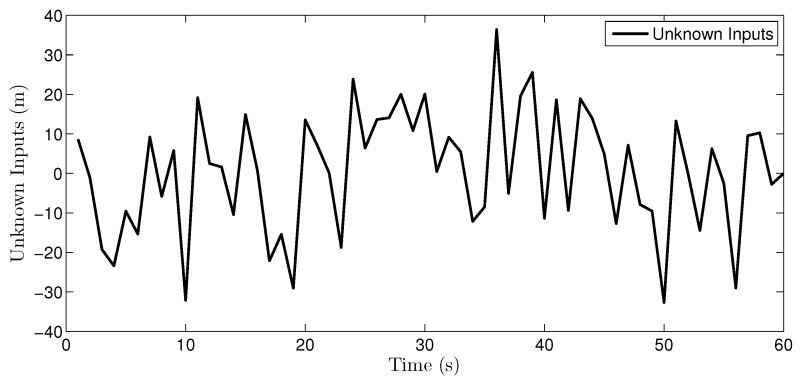
Real unknown input in the multi-sensor system of 60 sensors.

The UI, as plotted in Figure 12, is a stochastic noise obeying Gaussian distribution with mean 0 and covariance $15^{2}$.

**Figure 13:**
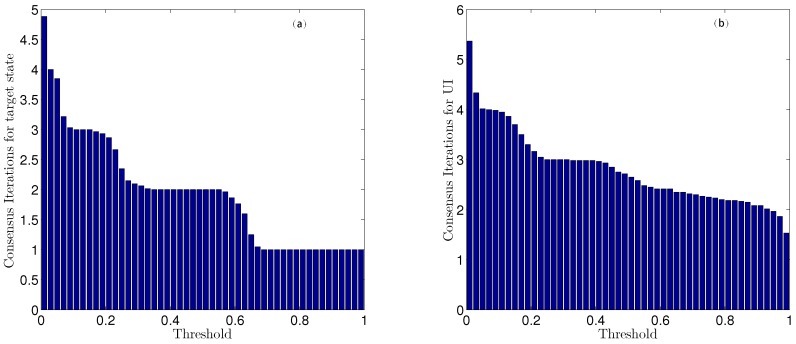
The mean numbers of iteration steps for (**a**) state and (**b**) UI estimation with different thresholds in the multi-sensor system of 60 sensors.

The histograms in Figure 13 depict the mean numbers of consensus iteration with different thresholds. Similarly, the consensus iteration number increases with thresholds decreasing, and when the thresholds are less than 0.1, the iteration step number increase sharply. Therefore, we choose the threshold as $\eta^{x}=\eta^{d}=$ 0.1. It is interesting to see from the histograms that the consensus iteration needed for UI is roughly one more time than that needed for sensor states.

**Figure 14:**
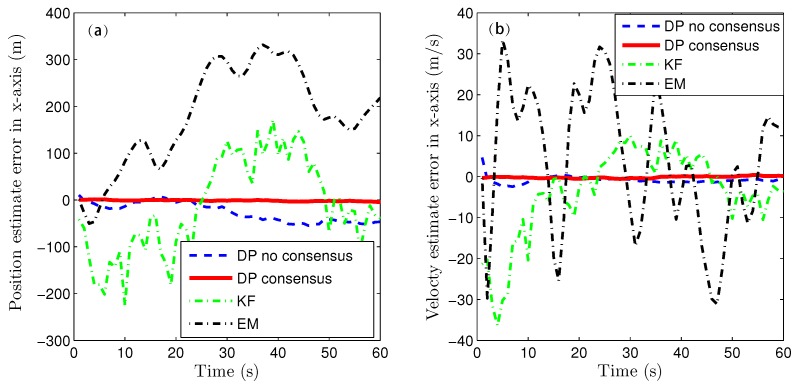
State estimation errors in the x direction using the KF, DP consensus, DP no consensus and EM methods for sensor 4 in the multi-sensor system of 60 sensors. (**a**) position estimation; (**b**) velocty estimation.

**Figure 15:**
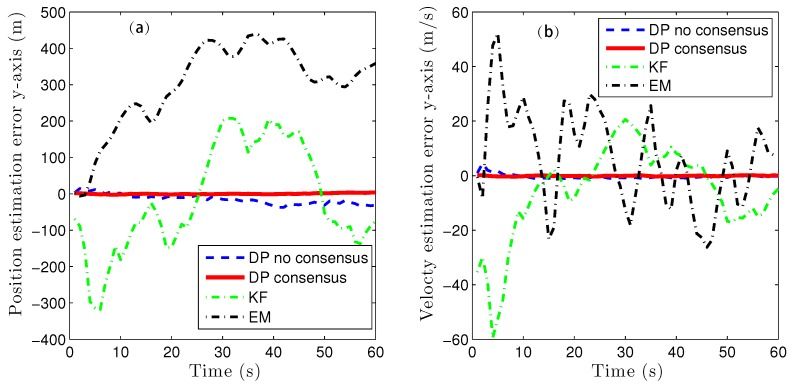
State estimation errors in the y direction using the KF, DP consensus, DP no consensus and EM methods for sensor 4 in the multi-sensor system of 60 sensors. (**a**) position estimation; (**b**) velocty estimation.

**Figure 16:**
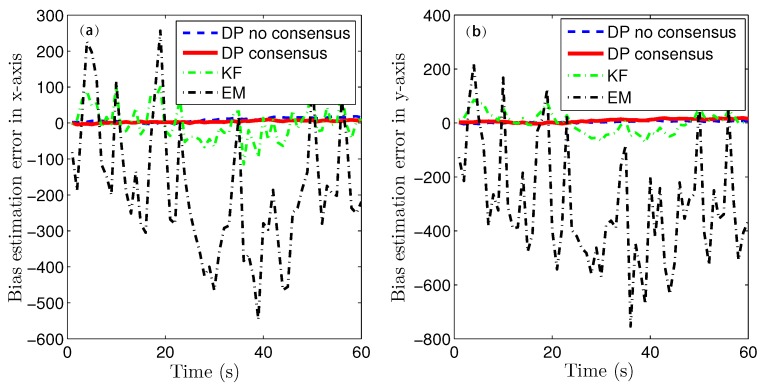
Bias estimation errors using the KF, DP consensus, DP no consensus and EM methods for sensor 4 in the multi-sensor system of 60 sensors. (**a**) x-axis; (**b**) y-axis.

**Figure 17:**
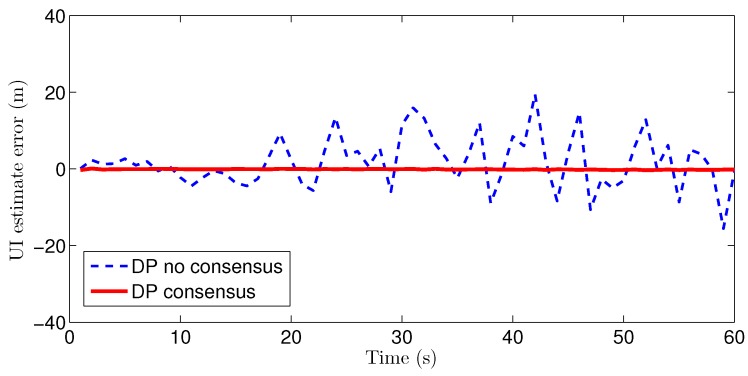
UI estimation errors of the DP consensus and DP no consensus methods for sensor 4 in the multi-sensor system of 60 sensors.

Due to the page limitation and the similar data variation trend in simulation figures, only the estimation results for the forth sensor is presented here. The iteration threshold for EM algorithm is $\delta =10^{-4}$ and the processing window length is l=5 too. Figure 14, Figure 15, Figure 16 and Figure 17 show the proposed algorithm estimation results are obviously better than those of KF and EM methods. Indeed, The estimation errors are greatly reduced by the DP consensus operation.

The mean and peak values of the estimation errors of the target state ${\left[x,\dot{x},y,\dot{y}\right]}^{T}$ and the bias ${\left[b_{x},b_{y}\right]}^{T}$ based on the KF, EM and DP methods are correspondingly computed and listed in Table 6 and Table 7, respectively. It can be seen from the Tables that our proposed method is proven to be effective.

The run times listed in Table 8 show that the run time of DP consensus is slightly shorter than that of EM method.

### 4.3. Computational Burden Analysis

Computation burden is also a critical issue to determine the practical applicability of the proposed method and worth to be discussed. Generally speaking, the computational burden is strongly related to the total sensors’ number and the connectivity of the sensor network. Thus, the mean values of run times of DP consensus method are tested with different sensor numbers and connectivity probabilities. In the simulation, the network are generated randomly with different connective probabilities. And the simulation program runs ten times with every connective probability and sensor number. The mean values of run time for one sensor of processing 60 samples are recorded to measure the computational burden.

**Figure 18:**
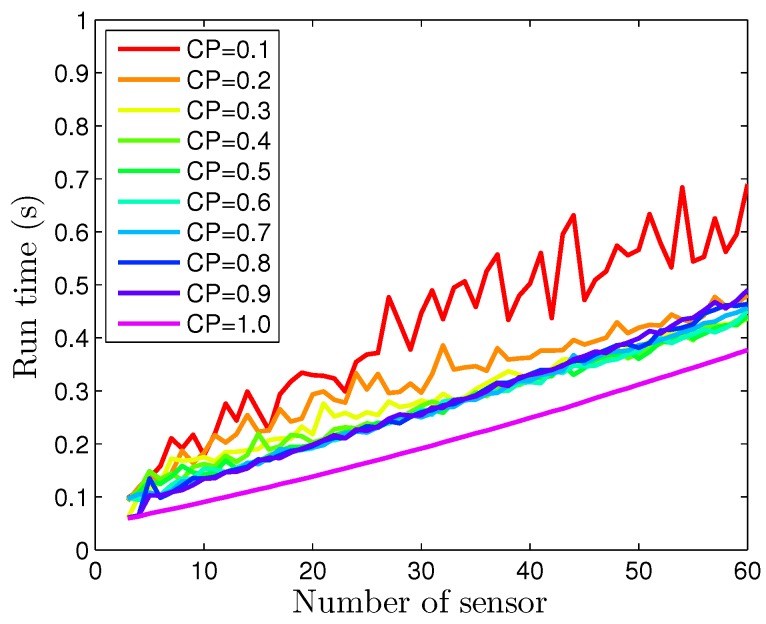
The mean values of the run times with different sensor numbers.

According to Figure 6 and Figure 13, the thresholds are chosen as $\eta^{x}=\eta^{d}=$ 0.1 in this simulation. Further, Figure 18 depicted the relationship between the mean of the run time and the number of sensor at different connectivity probabilities (’CP’ in Figure 18). It can be seen from Figure 18 that the run time increases with sensor number increase. It means more iterations are needed to reach a consensus when sensor number increase. Particularly, when the number of the sensor are 12 and 60 with connectivity probability of 1, the run times reach 0.08 s and 0.28 s, respectively. Furthermore, when the sensor number fixed, the run time increases with the connectivity probability decreases. This is because that a looser sensor network topology means fewer neighboring sensors, therefore, more jumpers are needed between each pair of sensors. So more iterations are needed to reach consensus, and the computational cost is corresponding high. It is also worth to mention that when the connectivity probability is larger than 0.2, run time almost have no difference. It implies that our proposed algorithm is somewhat robust when the connectivity probability is larger than 0.2.

As Figure 18 shows, with the sensor number increases, the mean values of run time for one sensor increases linearly. Thus, our proposed method can handle a relatively large network in a reasonable period of time.

## 5. Conclusions

The joint estimation of system state and sensor bias based on a generalized system model is proposed. The conditions for UI decoupling derived in this paper are helpful for guiding sensor choice and deployment. Local UI estimates are obtained via the LS approach using all estimates from neighboring sensors. Network consensus processing is then applied to obtain more accurate estimates of the system (target) state and the UI. Future work may address distributive and collaborative processing in sensor networks in the case in which bias exists in only a subset of the sensors.

## Figures and Tables

**Table 1:** Pseudo-code for the local MMSE estimation.

**Check the Applicability of the Method**
Step 1: Check the existence condition given by Equation (10). If the condition is satisfied, then perform the following computation. Otherwise, the proposed method is not applicable.
**Offline Equivalence Transformation**
Step 2: Construct the equivalent system-state evolution model by defining ${\bar{F}}_{k}^{i}$, $A_{k}^{i}$, ${\bar{C}}_{k}^{i}$, ${\bar{B}}_{k}^{i}$, and ${\bar{w}}_{k}^{i}$ using Equations (12)–(16).
Step 3. Determine the parameters $\Pi_{k}^{i}$ and $M_{k}^{i}$ using Equations (12) and (18).
**Online Estimator Implementation**
Step 4: Initialize the estimators: $P_{0|0}^{i}=P^{i}\left(0\right)$ and $z_{0}^{i}=z^{i}\left(0\right)$.
Step 5: Compute the predictions ${\hat{z}}_{k+1/k}^{i}$ and the corresponding covariances $P_{k+1|k}^{i}$ using Equations (19) and (20).
Step 6: Calculate the filter gains $K_{k+1}^{i}$ using Equation (23).
Step 7: Obtain the ${\hat{z}}_{k+1/k+1}^{i}$ and the corresponding covariances $P_{k+1|k+1}^{i}$ using Equations (21) and (22).
Step 8: Set k=k+1 and go to Step 1.

**Table 2:** Pseudo-code for the consensus estimation.

**Formulate the Local Information Before Transmission**
Step 1: Calculate the estimators ${\bar{b}}_{k}^{i}$ using Equation (24).
**Exchange Information with All Neighbours of the *i*th Sensor**
Step 2: Exchange $G_{k}^{i}$, ${\hat{x}}_{k|k}^{i}$, $P_{k|k}^{xx,i}$, and ${\bar{b}}_{k}^{i}$ with all neighbouring sensors.
**Formulate the Local LS Estimators of the UI**
Step 3: Define the parameters $G_{k}$ and $B_{k}$.
Step 4: Use the exchanged information to calculate ${\hat{d}}_{k-1}^{i}$ and $P_{k-1}^{dd,i}$ via Equations (25) and (26).
**Consensus Estimation after Transmission**
Step 5: Set l=1, ${\hat{x}}_{k|k}^{i}\left(0\right)={\hat{x}}_{k|k}^{i}$, $P_{k|k}^{xx,i}\left(0\right)=P_{k|k}^{xx,i}$, ${\hat{d}}_{k-1}^{i}\left(0\right)={\hat{d}}_{k-1}^{i}$ and $P_{k-1}^{dd,i}\left(0\right)=P_{k-1}^{dd,i}$.
Step 6: Calculate the ACF estimators ${\hat{x}}_{k|k}^{i}\left(l\right)$ and $P_{k|k}^{xx,i}\left(l\right)$ using Equations (27) and (29).
Step 7: Calculate the ACF estimators $d_{k-1}^{i}\left(l\right)$ and $P_{k-1}^{dd,i}\left(l\right)$ using Equations (28) and (30).
Step 8: If Equations (31) and (32) hold, go to Step 9; otherwise, set l=l+1 and go to Step 6.
**Refine the Local Sensor Bias Estimators after Consensus**
Step 9: Refine the estimates $b_{k|k}^{i}$ and $P_{k|k}^{i,bb}$ using Equations (33) and (34).
Step 10: Set k=k+1 and go to Step 1.

**Table 3:** Comparison of mean estimation error values.

	*x*	$\overset{˙}{x}$	*y*	$\overset{˙}{y}$	$b_{x}$	$b_{y}$
KF (m)	1.18	0.48	−2.66	0.29	1.16	−0.28
EM (m)	26.83	0.79	32.33	0.91	−6.89	5.76
DP no consensus (m)	−10.87	−0.26	18.81	0.63	2.92	−1.51
DP consensus (m)	0.19	0.09	−9.55	0.27	−1.37	0.39

**Table 4:** Comparison of peak estimation error values.

	*x*	$\overset{˙}{x}$	*y*	$\overset{˙}{y}$	$b_{x}$	$b_{y}$
KF (m)	76.27	6.21	48.52	3.76	19.11	17.07
EM (m)	62.26	10.81	59.95	10.30	19.84	12.33
DP no consensus (m)	30.55	1.71	46.73	4.56	9.47	8.12
DP consensus (m)	4.28	1.17	13.90	1.98	4.08	4.67

**Table 5:** Total run times (60 samples).

	EM	DP No Consensus	DP Consensus
time (s)	6.57	0.33	4.89

**Table 6:** Comparison of mean estimation error values of the network of 60 sensors.

	*x*	$\overset{˙}{x}$	*y*	$\overset{˙}{y}$	$b_{x}$	$b_{y}$
KF (m)	−12.721	−3.86	−13.54	−5.72	−0.40	1.19
EM (m)	180.14	4.16	299.97	6.58	−182.47	−306.07
DP no consensus (m)	−20.73	−0.84	−15.03	−0.34	8.56	3.63
DP consensus (m)	−1.21	−0.15	0.063	−0.09	3.63	8.56

**Table 7:** Comparison of peak estimation error values of the network of 60 sensors.

	*x*	$\overset{˙}{x}$	*y*	$\overset{˙}{y}$	$b_{x}$	$b_{y}$
KF (m)	396.46	80.08	526.54	80.03	220.43	156.67
EM (m)	381.98	64.60	443.66	78.49	803.40	973.76
DP no consensus (m)	65.62	7.12	52.05	5.54	21.61	14.26
DP consensus (m)	5.73	0.94	5.76	0.59	12.91	21.61

**Table 8:** Total run times (60 samples) of the network of 60 sensors.

	EM	DP No Consensus	DP Consensus
time (s)	27.39	1.65	24.56

## Abbreviations

UI	Unknown input
GSB	generalized sensor biases
EM	Expectation-maximization
IMM	Interacting multiple model
LMMSE	Linear minimum-mean-square-error
MUBF	Minimum upper bound filter
SB	Sensor bias
ECM	Electronic counter-measure
LSB	Local sensor bias
GLSB	Global sensor bias
ACF	Average consensus fusion
DLS	Distributed least squares
WSN	Wireless sensor networks
KF	Kalman Filter
DP no consensus	The proposed method without consensus processing
DP consensus	The proposed method with consensus processing
CP	Connectivity probability

## Appendix A

**Proof of Lemma 1.** Sufficient: If rank$\left(\Gamma \Psi \right)=$rank$\left(\Psi \right)$ and Ψ is of full column rank, then the matrix ΓΨ is also of full column rank, and hence, its left pseudo-inverse matrix ${\left(\Gamma \Psi \right)}^{+}={\left[{\left(\Gamma \Psi \right)}^{T}\left(\Gamma \Psi \right)\right]}^{-1}{\left(\Gamma \Psi \right)}^{T}$ exists, satisfying

(A1) $${\left(\Gamma \Psi \right)}^{+}\Gamma \Psi =I$$

Clearly, Equation (6) holds.

Necessary: If Equation (6) holds, then $\Psi =\Psi {\left(\Gamma \Psi \right)}^{+}\left(\Gamma \Psi \right)$ or $\Psi^{T}={\left(\Gamma \Psi \right)}^{T}{\left[\Psi {\left(\Gamma \Psi \right)}^{+}\right]}^{T}$, implying that $\Psi^{T}$ belongs to the space of the matrix ${\left(\Gamma \Psi \right)}^{T}$; hence, we have the following inequality:

$$\mathrm{rank}\left\{{\left(\Psi \right)}^{T}\right\}\leq \mathrm{rank}\left\{{\left(\Gamma \Psi \right)}^{T}\right\}$$

or

(A2) rank{Ψ}≤rank{ΓΨ}

Meanwhile, we know that

(A3) $$\mathrm{rank}\left\{\Gamma \Psi \right\}\leq \min\left(\mathrm{rank}\{\Gamma \},\;\mathrm{rank}\{\Psi \}\right)\leq \mathrm{rank}\left\{\Psi \right\}$$

Combining Equations (A2) and (A3), we obtain Equation (7). ☐

## Appendix B

**Proof of Theorem 2.** By left-multiplying both sides of Equation (8) by ${\bar{H}}_{k+1}^{i}$, we obtain

(B1) $${\bar{H}}_{k+1}^{i}z_{k+1}^{i}={\bar{H}}_{k+1}^{i}A_{k}^{i}z_{k}^{i}+{\bar{H}}_{k+1}^{i}{\bar{G}}_{k}^{i}d_{k}+{\bar{H}}_{k+1}^{i}{\bar{\zeta }}_{k}^{i}$$

Meanwhile, Equation (9) can be rewritten as

(B2) $${\bar{H}}_{k+1}^{i}z_{k+1}^{i}=y_{k+1}^{i}-v_{k+1}^{i}$$

Then, substituting Equation (B1) into Equation (B2) yields

$${\bar{H}}_{k+1}^{i}{\bar{G}}_{k}^{i}d_{k}=y_{k+1}^{i}-v_{k+1}^{i}-{\bar{H}}_{k+1}^{i}A_{k}^{i}z_{k}^{i}-{\bar{H}}_{k+1}^{i}{\bar{\zeta }}_{k}^{i}$$

with the following solution:

(B3) $$d_{k}=\Pi_{k}^{i}\left(y_{k+1}^{i}-v_{k+1}^{i}-{\bar{H}}_{k+1}^{i}A_{k}^{i}z_{k}^{i}-{\bar{H}}_{k+1}^{i}{\bar{\zeta }}_{k}^{i}\right)+\left[I_{q}-\Pi_{k}^{i}\left({\bar{H}}_{k+1}^{i}{\bar{G}}_{k}^{i}\right)\right]\kappa$$

where $\Pi_{k}^{i}≐{\left({\bar{H}}_{k+1}^{i}{\bar{G}}_{k}^{i}\right)}^{+}={\left[{\left({\bar{H}}_{k+1}^{i}{\bar{G}}_{k}^{i}\right)}^{T}{\bar{H}}_{k+1}^{i}{\bar{G}}_{k}^{i}\right]}^{-1}{\left({\bar{H}}_{k+1}^{i}{\bar{G}}_{k}^{i}\right)}^{T}$ in Equation (12) and Equation (B3) and κ is an arbitrary matrix with the proper dimensions.

Further substituting Equation (B3) into Equation (8) yields

(B4) $$\begin{array}{c} \begin{array}{c} z_{k+1}^{i}=A_{k}^{i}z_{k}^{i}+{\bar{\zeta }}_{k}^{i}+{\bar{C}}_{k}^{i}\left(y_{k+1}^{i}-v_{k+1}^{i}-{\bar{H}}_{k+1}^{i}A_{k}^{i}z_{k}^{i}-{\bar{H}}_{k+1}^{i}{\bar{\zeta }}_{k}^{i}\right)+{\bar{G}}_{k}^{i}\left(I_{q}-\Pi_{k}^{i}{\bar{H}}_{k+1}^{i}{\bar{G}}_{k}^{i}\right)\kappa \\ \;\;\;\;\;\;\;\;={\bar{F}}_{k}^{i}A_{k}^{i}z_{k}^{i}+{\bar{C}}_{k}^{i}y_{k+1}^{i}+{\bar{B}}_{k}^{i}{\bar{w}}_{k}^{i}+{\bar{G}}_{k}^{i}\left[I_{q}-\Pi_{k}^{i}{\bar{H}}_{k+1}^{i}{\bar{G}}_{k}^{i}\right]\kappa \end{array} \end{array}$$

where ${\bar{C}}_{k}^{i}$ and ${\bar{B}}_{k}^{i}$ are given in Equations (13) and (16), respectively. For any *κ*, the necessary and sufficient condition for guaranteeing the uniqueness of $z_{k+1}^{i}$ in Equation (B4) is

(B5) $${\bar{G}}_{k}^{i}\left(I_{q}-\Pi_{k}^{i}{\bar{H}}_{k+1}^{i}{\bar{G}}_{k}^{i}\right)={\bar{G}}_{k}^{i}\left(I_{q}-{\left({\bar{H}}_{k+1}^{i}{\bar{G}}_{k}^{i}\right)}^{+}{\bar{H}}_{k+1}^{i}{\bar{G}}_{k}^{i}\right)=0$$

According to Lemma 1, Equation (B5) will hold if and only if Equation (10) holds. Finally, substituting Equation (B5) into Equation (B4) results in Equations (11)–(13). ☐

## Appendix C

**Proof of Theorem 4.** In Equation (27), $w_{ij}^{x}\left(l\right)\geq 0$ denotes the probability of being in state i at iteration l. The covariance of the mixture is

(C1) $$\begin{array}{ccc} & & E\left\{\left[x_{k}-{\hat{x}}_{k|k}^{i}\left(l+1\right)\right]{\left[x_{k}-{\hat{x}}_{k|k}^{i}\left(l+1\right)\right]}^{T}\right\}\\ & = & \underset{j\in J_{i}}{E\sum }w_{ij}^{x}\left(l\right)\left\{\left[x_{k}-{\hat{x}}_{k|k}^{j}\left(l\right)+{\hat{x}}_{k|k}^{j}\left(l\right)-{\hat{x}}_{k|k}^{i}\left(l+1\right)\right]{\left[x_{k}-{\hat{x}}_{k|k}^{j}\left(l\right)+{\hat{x}}_{k|k}^{j}\left(l\right)-{\hat{x}}_{k|k}^{i}\left(l+1\right)\right]}^{T}\right\}\\ & = & \underset{j\in J_{i}}{E\sum }w_{ij}^{x}\left(l\right)\left\{\left[\left(x_{k}-{\hat{x}}_{k|k}^{j}\left(l\right)\right){\left(x_{k}-{\hat{x}}_{k|k}^{j}\left(l\right)\right)}^{T}\right]+\left[{\hat{x}}_{k|k}^{j}\left(l\right)-{\hat{x}}_{k|k}^{i}\left(l+1\right)\right]{\left[{\hat{x}}_{k|k}^{j}\left(l\right)-{\hat{x}}_{k|k}^{i}\left(l+1\right)\right]}^{T}\right\}\\ & = & \sum_{j\in J_{i}}w_{ij}^{x}\left(l\right)P_{k|k}^{xx,i}\left(l\right)+\sum_{j\in J_{i}}w_{ij}^{x}\left(l\right)\left[{\hat{x}}_{k|k}^{j}\left(l\right)-{\hat{x}}_{k|k}^{i}\left(l+1\right)\right]{\left[{\hat{x}}_{k|k}^{j}\left(l\right)-{\hat{x}}_{k|k}^{i}\left(l+1\right)\right]}^{T} \end{array}$$

The second term in Equation (C1) has the following alternative expression:

(C2) $$\begin{array}{c} \begin{array}{cc} & \sum_{j\in J_{i}}w_{ij}^{x}\left(l\right)\left[{\hat{x}}_{k|k}^{j}\left(l\right)-{\hat{x}}_{k|k}^{i}\left(l+1\right)\right]{\left[{\hat{x}}_{k|k}^{j}\left(l\right)-{\hat{x}}_{k|k}^{i}\left(l+1\right)\right]}^{T}\\ = & \sum_{j\in J_{i}}w_{ij}^{x}\left(l\right){\hat{x}}_{k|k}^{j}\left(l\right){\left({\hat{x}}_{k|k}^{j}\left(l\right)\right)}^{T}-{\hat{x}}_{k|k}^{i}\left(l+1\right){\left({\hat{x}}_{k|k}^{i}\left(l+1\right)\right)}^{T} \end{array} \end{array}$$

Substituting Equation (C2) into Equation (C1) yields Equation (29), and Equation (30) can be obtained similarly. ☐
